# Fully Automated Evaluation of Left Ventricular Global Longitudinal Strain in Normal Hearts: An Assessment of Accuracy in the Pediatric Population

**DOI:** 10.1007/s00246-025-04074-2

**Published:** 2025-11-17

**Authors:** Hunter Kauffman, Michael J. Convery, Daisuke Matsubara, Karen E. Miller, Matthew J. O’Connor, Michael D. Quartermain, Anirban Banerjee

**Affiliations:** Division of Cardiology, Children’s Hospital of Philadelphia, 3401 Civic Center Blvd, Philadelphia, PA 19104 USA

**Keywords:** Echocardiography, Pediatric cardiology, Strain imaging, Fully automated analysis

## Abstract

Two-dimensional echocardiography has been shown to be a useful modality for analyzing cardiac deformation parameters. However, the clinical utility of strain analysis is limited by time and training required for manual border tracing. This study aims to assess the feasibility of a “hands-off,” measurement program, Philips AutoStrain, for measurements of left-ventricular deformation in children. We hypothesize that AutoStrain may accurately calculate deformation measurements, but its performance may be limited in certain patient populations. Echocardiographic images from 168 patients were analyzed via AutoStrain using fully-automated and semiautomated approaches. GLS were manually traced offline as a reference standard. Autostrain outputs were analyzed for accuracy using percent difference from the reference standard and an accuracy scoring system designed to objectively quantify the precision of Autostrain border tracings at the apex and mitral annuli. Both fully-automated and semiautomated AutoStrain correlated well with manual GLS (Fully-automated *R* = 0.71, Semiautomated *R* = 0.90). However, the accuracy of AutoStrain was affected by image axis, patient age, and clear visualization of the LV apex. Fully-automated techniques may lead to a “hands-off” future in assessment of LV deformation in older children, but should be used cautiously in younger children until further refinements in the algorithm are performed.

**Supplementary Information:**

The online version contains supplementary material available at https://doi.org/10.1007/s00246-025-04074-2.

## Introduction

The quantification of left ventricular (LV) deformation has become a valuable modality for evaluating cardiac function in the pediatric population. Accurate assessment of LV function is critical in diagnosing, managing, and monitoring a wide array of cardiovascular conditions in children [1–3]. Among the available imaging modalities, transthoracic echocardiography (TTE) remains the most commonly employed tool due to its noninvasive nature, accessibility, and cost-effectiveness [4]. Specifically, 2-dimensional echocardiography (2DE) is widely utilized in clinical practice to assess LV size and function. Despite its advantages, 2DE may be limited by factors such as image quality, dependency on operator experience, and interobserver variability [5–8]. 

Global longitudinal strain (GLS) derived from 2DE has emerged as a sensitive and reproducible metric for assessing cardiac function [9, 10]. GLS measures the longitudinal deformation of myocardial fibers, providing valuable insights into subclinical cardiac dysfunction that may not be detected by more conventional metrics, such as ejection fraction [1, 3, 6, 7]. However, strain measurement accuracy can vary significantly depending on the technique used. Variability may arise from differences in vendor-specific versus vendor-independent software, underlying speckle-tracking algorithms, operator experience, and image quality [5–8]. These factors can influence both reproducibility and absolute strain values, creating challenges when interpreting results across platforms or studies.

In recent years, advancements in echocardiographic technology have introduced novel software programs capable of automating GLS measurements [11–14]. These innovations aim to enhance the accuracy and reproducibility of strain analysis while reducing interobserver variability and time consumption. AutoStrain, a 2DE-based software program, which utilizes speckle tracking to calculate GLS, has been validated in adults for providing precise assessments of LV strain and function [15]. It has also shown promise in older children, suggesting its potential for broader application in pediatric populations [16]. However, there is a paucity of data evaluating the accuracy, reproducibility, and limitations of AutoStrain in younger pediatric populations, where factors such as smaller heart size, higher heart rates, and variable acoustic windows may pose unique challenges [17]. 

Given the critical importance of accurate GLS measurements in pediatric patients, our study seeks to address this gap in knowledge. Specifically, the aims of this investigation are: (1) To assess the accuracy and efficacy of AutoStrain software in a pediatric population compared to manual, offline strain analysis, which remains the current gold standard. (2) To identify patient or technical factors that may limit the ability of AutoStrain to produce reliable and accurate GLS outputs.

We hypothesize that fully automated AutoStrain GLS measurements will demonstrate strong accuracy and robust reliability compared with manual GLS tracing using TomTec software. As a secondary aim, we seek to evaluate whether patient age and off-axis imaging introduce inaccuracy, and to identify specific left ventricular regions that are unreliably tracked by the AutoStrain software.

## Methods

### Study Design and Population

This is a prospective, validation study assessing the clinical utility of AutoStrain. We included data from 170 patients who underwent TTE at the Children’s Hospital of Philadelphia from February 2020 to April 2021. All images were obtained at bedside without sedation during outpatient echocardiography exams. Patients with LVEF < 55%, single ventricles, and other complex congenital anomalies were excluded. For all patients, demographic data were abstracted via medical chart review. This study was approved by the Institutional Review Board of The Children’s Hospital of Philadelphia, and all participants provided written informed consent before acquiring the research images.

### Acquisition and Analysis of Echocardiographic Images

Echocardiographic images were acquired by experienced sonographers using the EPIQ CVx ultrasound systems equipped with X5 transducers (Philips Medical Systems, Andover, MA, USA). The imaging protocol included standardized acquisition of 2D images in the apical four-chamber (4Ch), two-chamber (2Ch), and three-chamber (3Ch) views. Sonographers carefully optimized each image to minimize acoustic artifacts and achieve clear visualization of the endocardial borders, and LV apex. All images were acquired following best practices to ensure reproducibility and diagnostic quality. In addition, 14 deliberate, “off axis”, acquisitions were obtained to test the performance of AutoStrain under suboptimal imaging conditions, where the LV apex may not be well visualized. These 14 images were not included in data analysis.

After acquisition, the 2DE images were electronically stored in the system’s database and exported for offline analysis using Syngo Dynamics software (Siemens, Munich, Germany). Images were exported at their native frame rate, preserving temporal and spatial resolution for consistency and accuracy in later analyses.

**Fig. 1:**
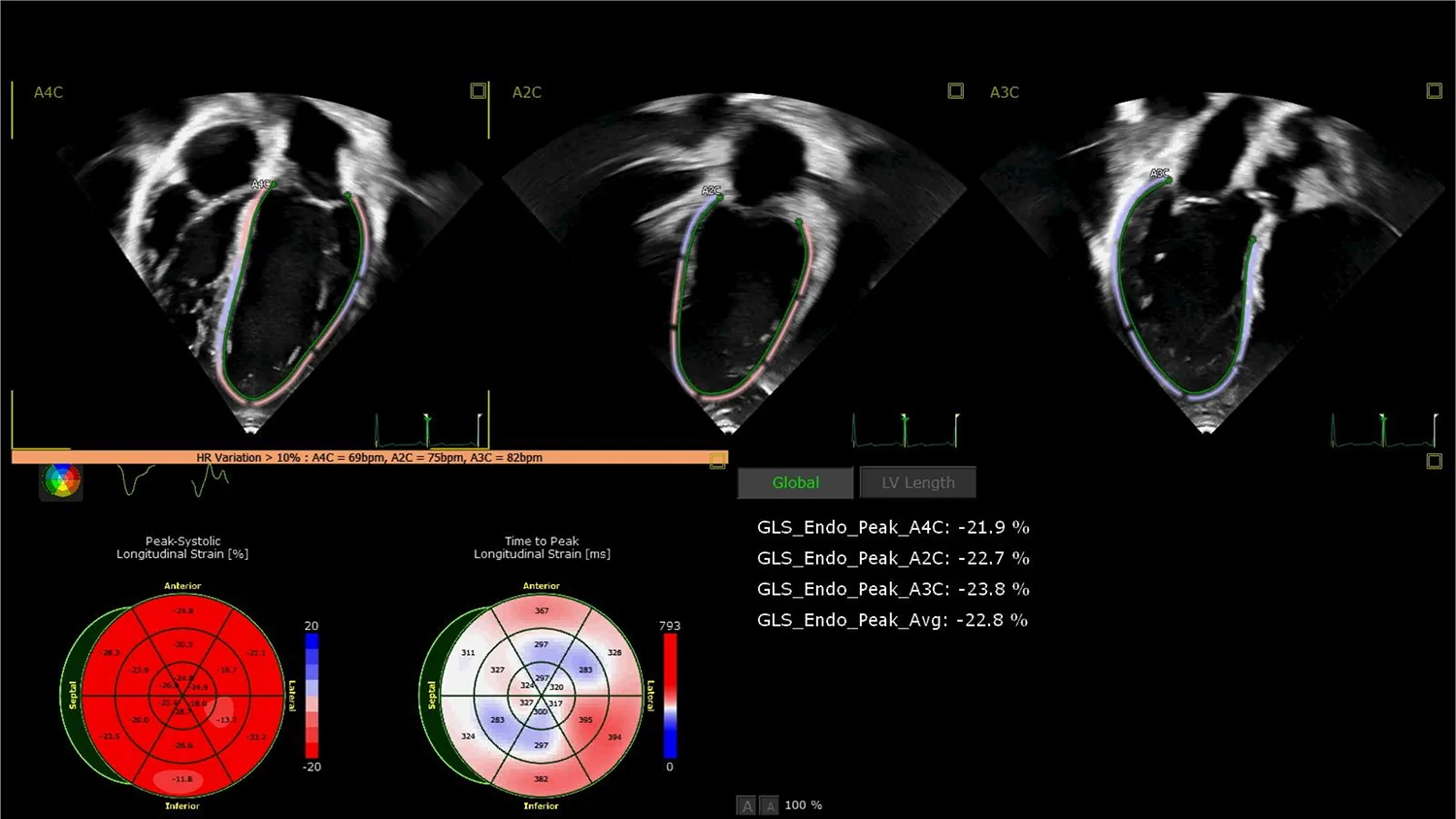
Representative output from 2D AutoStrain analysis. The automated contour detection outlines the left ventricular endocardial borders and generates longitudinal strain curves for each myocardial segment. The figure provides a combined visual summary of global longitudinal strain (GLS) and regional segmental strain values, allowing quick assessment of strain distribution across the ventricle

At the bedside, AutoStrain analysis was performed directly on the EPIQ CVx system via a single button press. For each patient, a single, unedited AutoStrain output was generated and stored as the initial assessment. In cases where automated tracking required refinement, manual editing of the AutoStrain results was performed to improve the accuracy of strain measurements. (Fig. 1) Moreover, the time taken to analyze both fully automated and semiautomated GLS was recorded by an investigator. This process allowed for evaluation of the efficiency and accuracy of both fully automated and semiautomated approaches.

Following the completion of imaging, all relevant data, including measurements and demographic variables, were abstracted and recorded into digital spreadsheets to facilitate statistical analysis. Additionally, 2DE images were exported for manual offline strain analysis, ensuring that manual GLS measurements could be directly compared with automated and semiautomated methods.

As a result, echocardiographic images were analyzed using three distinct methods: (1) Fully automated AutoStrain: A completely hands-off approach using the initial unedited AutoStrain output. (2) AutoStrain with manual editing: A semiautomated approach incorporating manual adjustments to improve tracking accuracy when necessary. (3) Manual GLS measurements: Offline analysis performed manually using exported images to provide an independent reference.

### Manual 2D Global Longitudinal Strain

Two-dimensional speckle-tracking echocardiographic analysis was conducted offline using vendor-independent software (2DCPA 1.3.0.91, TomTec Imaging Systems, Munich, Germany). The same 2D echocardiographic images used for AutoStrain analysis—apical 2Ch, 3Ch, and 4Ch views—were utilized to ensure consistency in image quality and acquisition parameters. Adequate image quality was determined by the clarity of endocardial border visualization throughout the cardiac cycle, absence of significant artifact or dropout, and optimal gain and focus settings, and adequately visualized LV segments. An experienced operator initiated the analysis by selecting the end-diastolic (ED) and end-systolic (ES) frames for each apical view. The ED frame was defined as the first frame following mitral valve closure, while the ES frame was identified as the point of maximal ventricular contraction, typically coinciding with the smallest LV cavity size. Endocardial borders were then manually traced in each view, ensuring precise delineation of the LV boundary. However, care was taken to exclude trabeculations and papillary muscles from the tracing to avoid skewing the strain measurements [18].

GLS was calculated as the average global shortening of the manually traced endocardial borders across all three apical views. The accuracy and reproducibility of this manual method served as the reference standard for comparison with AutoStrain-derived GLS values [19].

To evaluate the reliability of manual 2D GLS measurements, inter- and intraobserver variability assessments were performed in a subset of 25 randomly selected patients. For interobserver variability, two independent investigators (HK and DM) analyzed the same images separately. For intraobserver variability, one investigator (HK) repeated the analysis approximately three weeks after the initial measurements. Variability was quantified using intraclass correlation coefficients (ICC), providing a robust measure of the consistency of manual tracing techniques between and within observers.

### Border Tracking Assessment

A binary scoring system was developed to heuristically assess AutoStrain’s border-tracing accuracy, particularly in cases where AutoStrain outputs were normal despite inaccuracy of the tracing on visual inspection. Accuracy was evaluated at three anatomical landmarks—the lateral mitral annulus, medial mitral annulus, and LV apex—in the apical 2-chamber, 3-chamber, and 4-chamber views, at both end-systole (ES) and end-diastole (ED), resulting in 18 assessment points per patient. At each point, the automated contour was classified as a “hit” (score = 1) if it accurately aligned with the anatomical landmark without requiring manual adjustment, or a “miss” (score = 0) if it deviated significantly and required correction. Scores were summed to generate a global composite accuracy score (range 0–18). Regional performance was also evaluated by calculating the percentage of “hits” for each landmark across all patients, allowing identification of specific areas where AutoStrain performed well or had difficulty tracking borders.

### Age Group and Comparisons

To assess the accuracy of AutoStrain across different pediatric populations, we compared the percent difference from the reference standard GLS measurements between four distinct age groups: 0–5 years, 5–10 years, 10–15 years, and 15–18 years [20]. Percent difference was calculated by comparing AutoStrain-derived GLS values to the manual GLS measurements, which served as the reference standard. This comparison allowed us to evaluate how AutoStrain performed across varying developmental stages and anatomical differences between these age groups.

### Statistics

Correlations between automated AutoStrain and semiautomated AutoStrain with the reference standard were independently assessed using intraclass correlation coefficients (ICC) and simple linear regression. Bland–Altman analysis was performed to evaluate bias between methods. Data normality was assessed using the Shapiro–Wilk test. Comparisons of GLS percent difference and AutoStrain Accuracy Score between age groups were performed using one-way ANOVA. Inter- and intra-observer variability for manual GLS measurements were expressed as ICC values. For all analyses, a two-tailed p-value < 0.05 was considered statistically significant.

## Results

**Fig. 2:**
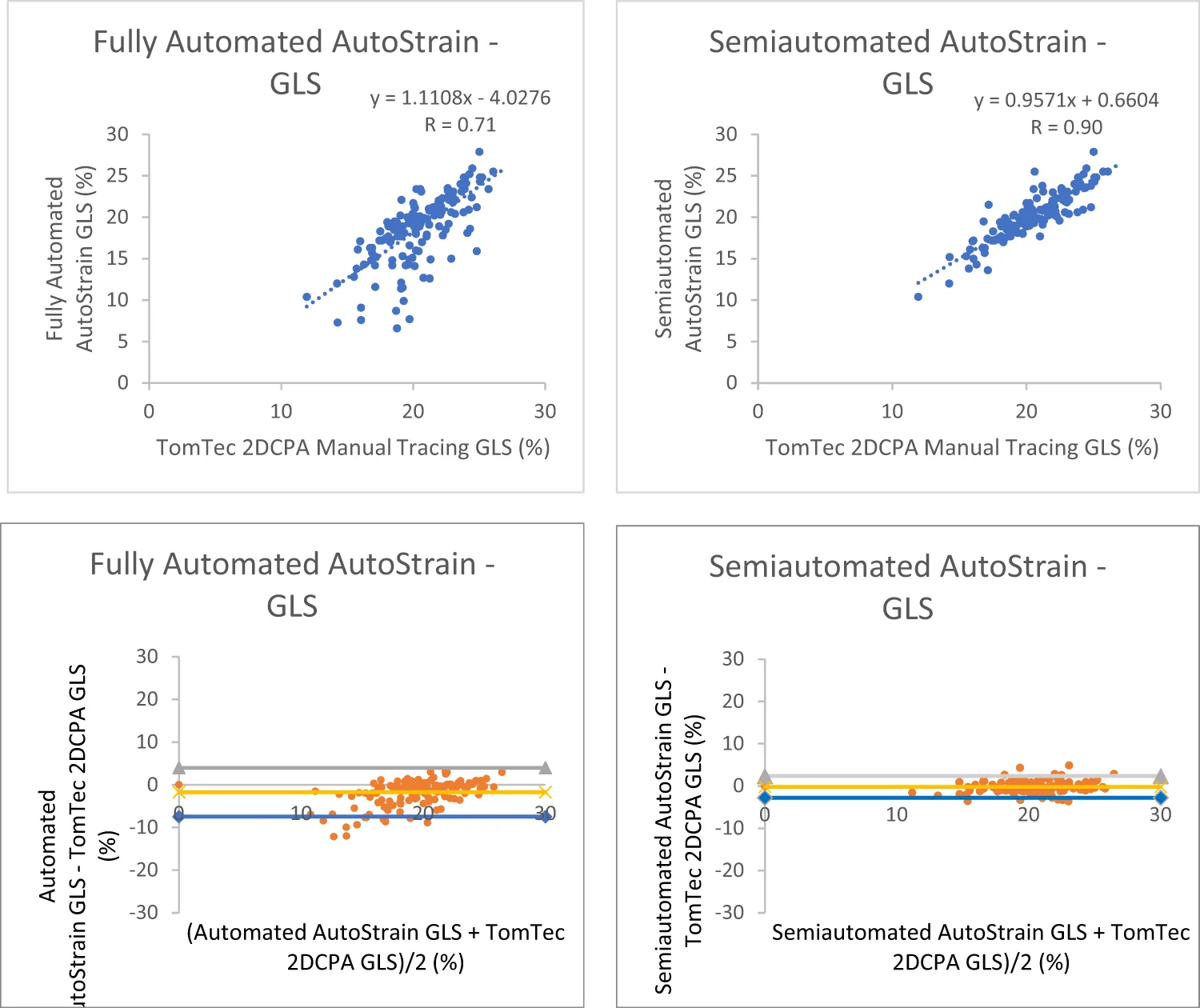
Regression and Bland–Altman analyses comparing automated and manual GLS measurements. This panel shows the correlation between GLS values obtained by fully automated AutoStrain, semiautomated AutoStrain, and the manual reference standard. Regression equations are displayed, with trendlines fitted without forcing the intercept through zero. The Bland–Altman plots demonstrate a small negative bias in AutoStrain-derived GLS relative to manual GLS, indicating that AutoStrain tends to slightly underestimate strain magnitude

**Fig. 3:**
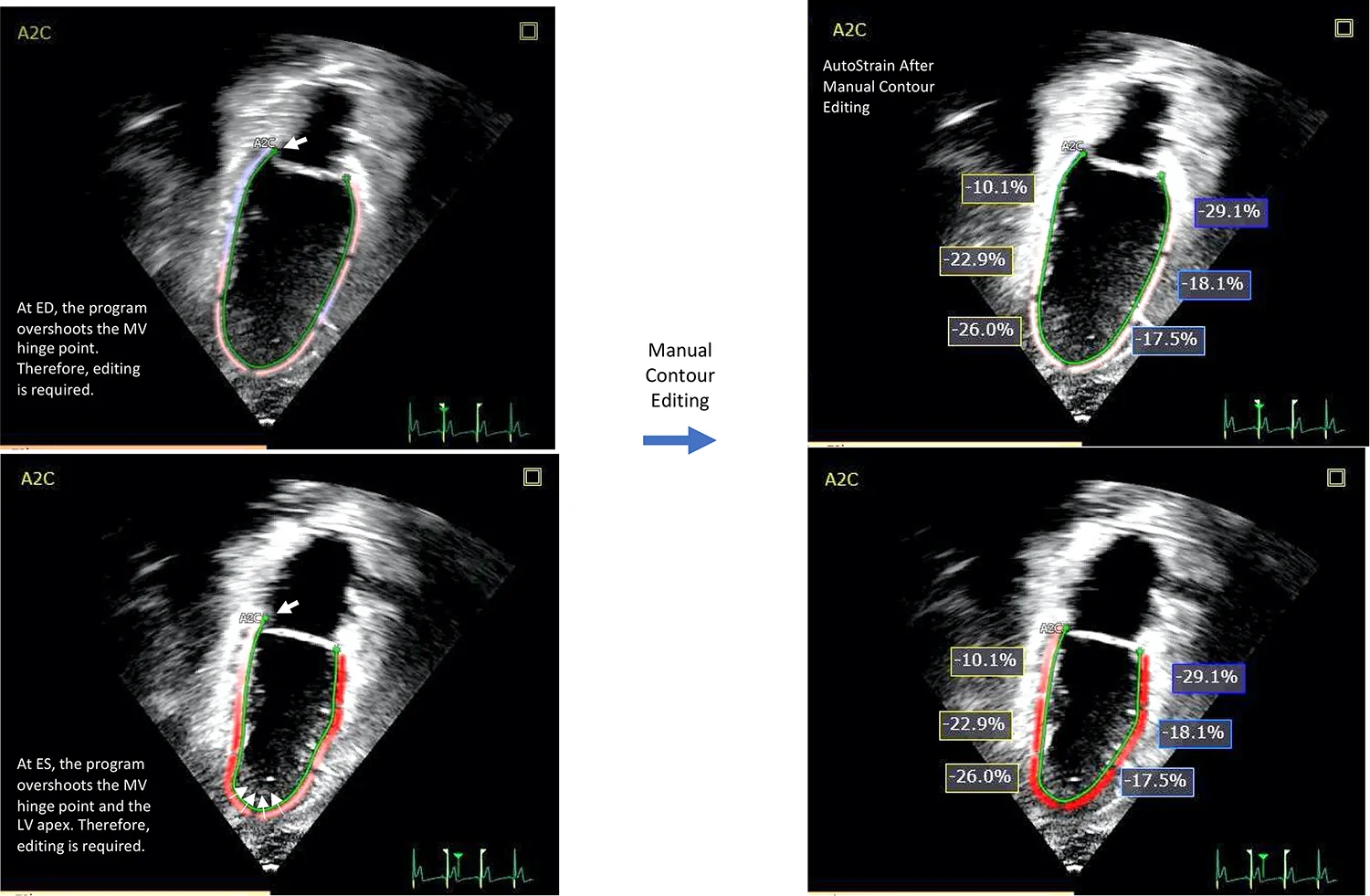
Example of semiautomated AutoStrain tracking adjustments. The white dashed lines depict the manually verified location of the true endocardial border. In the end-diastolic frame, the blue contour marks the epicardial border as initially placed by the automated software, illustrating the discrepancy that required manual correction during the semiautomated workflow

168 patients met inclusion criteria for this study, and 2 patients were excluded due to LVEF < 55%. Demographic data are presented in Table 1, and the results of AutoStrain and conventional echocardiographic analyses are summarized in Table 2. All images acquired for this study were of adequate diagnostic quality. AutoStrain automatically calculated GLS in an average time of 13 ± 4 s and showed a moderate correlation with manual GLS (*R* = 0.71, [95%CI 0.63–0.78] *p* < 0.05) on linear regression. After minor manual adjustments (average number of adjustments = 4), AutoStrain’s results showed a significantly stronger correlation with manual GLS (*R* = 0.90 [95%CI 0.87–0.93], *p* < 0.05)on linear regression. (Table 2; Fig. 2). However, editing increased the average analysis time to 73 ± 53 s, which was not statistically different from the manual method (81 ± 34 s, *p* = 0.22). Bland-Altman analysis revealed a slight negative bias (−1.7%) in AutoStrain compared to TomTec 2DCPA software, showing that the software tended to underestimate GLS. Moreover, this negative bias persisted in semiautomated AutoStrain (−0.2%) (Fig. 3). The average difference between AutoStrain-derived GLS with and without contour adjustments was 1.5 ± 3.6%. Reference standard GLS measurements using TomTec 2DCPA showed strong reliability, with inter- and intra-observer intraclass correlation coefficients (ICC) of 0.95 and 0.89, respectively.

**Table 1:** Cohort characteristics

Demographics	AutoStrain cohort *N* = 168	Shapiro-Wilks Test *p* value
Sex, Female (%)	38	0.21
Age (Years)	11.3 ± 4.5	0.09
Systolic BP (mmHg)	111 ± 12	0.38
Diastolic BP (mmHg)	63 ± 9	0.41
Height (cm)	147 ± 24	0.13
Weight (kg)	47 ± 23	0.07
BSA (m^2^)	1.37 ± 0.43	0.12
HR (BPM)	78 ± 19	0.32
BiPlane EF (%)	62 ± 4	0.43
Fully Automated GLS (%)	19.64 ± 3.27	0.27
Semiautomated GLS (%)	20.47 ± 2.77	0.06
Manual GLS (%)	20.86 ± 2.64	0.56

*BP* blood pressure, *BSA* body surface area, *HR* heart rate, *BPM* beats per minute, *EF* ejection fraction, *GLS* global longitudinal strain

**Table 2:** AutoStrain measurements vs. Reference standard

	AutoStrain	Manual strain	AutoStrain Time (s)	*R*	
Fully automated GLS (%)	19.64 ± 3.27	20.86 ± 2.64	13 ± 4	0.71	
Semiautomated GLS (%)	20.47 ± 2.77	20.86 ± 2.64	73 ± 53	0.90	

*TomTec 2DCPA* 2-dimensional cardiac performance analysis, *GLS* global longitudinal strain

### AutoStrain Border Tracking

**Fig. 4:**
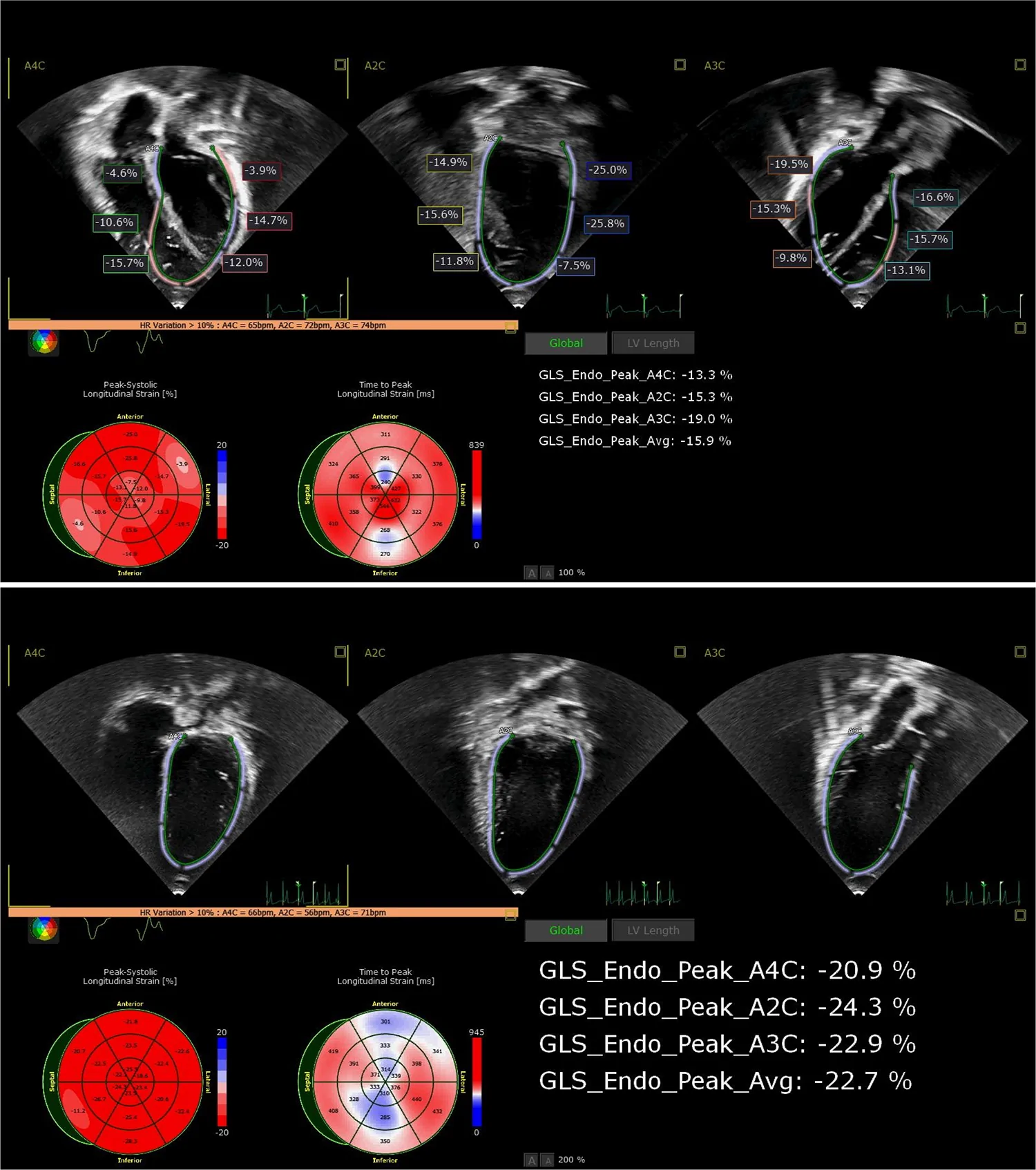
Impact of off-axis imaging on fully automated AutoStrain border detection. This example highlights how suboptimal apical view acquisition—particularly off-axis imaging—can cause misplacement of the automatically generated endocardial border, reducing GLS measurement accuracy in the fully automated approach

Table 3 summarizes the results of our system for assessing AutoStrain’s border-tracking accuracy. In this study, 36 of 168 patients (21%) required contour editing. The average composite accuracy scores for fully automated and semiautomated AutoStrain were 15.3 and 18, respectively. Notably, AutoStrain required adjustments at the apex more frequently than at the mitral annuli (Table 3; Fig. 4). In addition, the percent difference was significantly higher (*p* < 0.05), and the composite accuracy score was lower (*p* < 0.05) in the 0–5 age group compared to the other age groups (Table 4).

**Table 3:** Results of regional tracking analysis

Traces that required manual contour editing									
	4Ch view			3Ch view			2Ch view		
	Septal annulus	Lateral annulus	Apex	Septal annulus	Lateral annulus	Apex	Septal annulus	Lateral annulus	Apex
AutoStrain ED	7%	10%	8%	9%	9%	7%	8%	8%	9%
AutoStrain ES	12%	17%	20%	16%	16%	22%*	14%	15%	24%*

*ES* end-systole, *ED* end-diastole, *=*p* < 0.05

**Table 4:** Results of global tracking between age groups

Age group (number)	Frame rate (FPS)	Heart rate (BPM)	FR/HR ratio	GLS percent difference	Average A utoStrain Accuracy Sc ore
0–5 Years (*n* = 35)	58	99*	0.54*	18.6 ± 1.9*	4.7 ± 5.2*
5–10 Years (*n* = 44)	55	85	0.65	10.2 ± 3.5	13.3 ± 6.8
10–15 Years (*n* = 69)	52	77	0.64	7.3 ± 3.2	17.0 ± 2.9
15–18 Years (*n* = 34)	53	71	0.70	8.2 ± 2.9	17.1 ± 2.5

*GLS* global longitudinal strain, *FPS* frames per second, *BPM* beats per minute, *FR* frame rate, *HR* heart rate, *p < 0.05

### Off-Axis Images

In fourteen patients, additional “off-axis” images were obtained by sliding the probe more medially than the standard apical window. Upon analysis of these images, AutoStrain qualitatively recognized the two points around the mitral annulus but ubiquitously failed to track the LV apex in both systolic and diastolic phases across all three views (Fig. 4).

## Discussion

We found that GLS measurements obtained with the fully automated AutoStrain software in a cohort of pediatric patients with structurally normal hearts, showed moderate correlation with conventional GLS measurements. However, it became evident that manual contour adjustments were necessary to significantly enhance the correlation between AutoStrain and manual strain. While this process improved accuracy, it also came at the expense of increased analysis time. These findings underscore the potential of AutoStrain to offer clinically relevant automated measurements, even within pediatric populations, but also emphasize the critical importance of manual adjustments for optimal accuracy, particularly in younger or more challenging patient populations.

Several studies conducted in adults have demonstrated the feasibility of GLS measurements using speckle tracking-based algorithms, with the intent to automate strain analysis. However, the agreement of these automated algorithms with reference standards has shown variable results across different studies [21]. Additionally, many of the algorithms employed in previous research have been exclusively semi-automated, requiring manual corrections to improve the reliability of strain measurements and volumetric data [12–14]. In our study, we observed that the fully automated analysis of GLS using AutoStrain exhibited exceptionally high reproducibility. This is in line with findings from previous research, where fully automated algorithms demonstrated minimal inter- and intra-observer variability, a hallmark of automated systems [11]. This feature may be beneficial in echocardiography laboratories, where multiple readers or technicians may be involved in analyzing echocardiograms, as it can help standardize measurements and reduce variability [22]. 

Our results also demonstrate that AutoStrain sometimes required manual correction to improve its correlation with reference standards. Specifically, 21% of patients in our study required manual contour adjustments. When these corrections were not applied, the results of our Bland-Altman analysis revealed that GLS was underestimated by an average of 1.7%. However, in cases where GLS is near normal, this underestimation can be significant, potentially leading to an increased risk of type I error or false-positive results.

AutoStrain’s tracking analysis showed no significant relationship between the need for contour editing and patient size. However, it is essential to note that while GLS is a unitless, relative measure of cardiac deformation, it is not clear whether vendor-dependent software programs consider cardiac size or geometry. This potential limitation is particularly noteworthy in pediatric cardiology, where cardiac size, heart rate, and morphology can vary greatly. With this in mind, it is noteworthy that the percent difference and composite accuracy score of our 0–5 age group was significantly lower than other age groups. While the reason for this finding is not clear, we speculate that it may be related to interactions between speckle tracking software and the higher heart rates—and subsequently reduced frame rate to heart rate ratios—commonly seen in younger children [17]. This is supported by our finding of significantly increased HR and decreased FR/HR ratios seen in the 0–5 age group. (Table 4) Specifically, when the frame rate is too low relative to the heart rate, the natural acoustic markers within the myocardium used for tracking may shift excessively between consecutive frames. This excessive movement may make it difficult for the software to reliably identify and follow the same speckles throughout the cardiac cycle, leading to inaccuracies in strain measurement. In addition to frame rate effects, the smaller cardiac dimensions in younger patients may reduce the number of speckles available for tracking and limit spatial resolution, increasing susceptibility to signal-to-noise issues and reducing algorithm accuracy [17, 21]. 

In addition to the difficulties described in our youngest age group, our regional tracking analysis revealed that the LV apex required more frequent adjustments compared to other regions of the left ventricle, with more adjustments required in ES than ED. We speculate that the higher frequency of apex corrections in ES compared with ED likely reflects increased myocardial thickening, foreshortening, and endocardial displacement at peak contraction, which can challenge speckle-tracking algorithms more than the relatively stable borders seen in diastole [21]. These findings highlight the importance of a clear visualization of the LV apex, which is a critical component of accurate AutoStrain analysis. The significance of this was further corroborated by our observations in 14 deliberate “off-axis” image acquisitions, where AutoStrain successfully recognized the mitral annulus but universally failed to track the shifted LV apex. This underlines the importance of using well-aligned images, especially in clinical settings where patients may have anatomical challenges, such as those with a dilated right ventricle that alters the position of the apex or abnormal ventricular septal alignment.

As echocardiography continues to expand in use, especially for rapid bedside assessments in emergency departments and intensive care units, the need for automated tools that provide reliable, consistent measurements will only grow. As it stands, AutoStrain remains fast and reproducible. However, significant inconsistencies in border tracking and frequent need for contour adjustment limit the usefulness of this program for large scale population trials e.g. outcome trials. Moreover, these findings highlight the importance of careful operator oversight when using automated measurements in clinical decision making.

In pediatrics, AutoStrain remains limited by the lack of large-scale validation and its reliance on adult-trained algorithms. Fully automated GLS demonstrates accuracy when image quality is high and the apex is well visualized, though variability persists in younger children. AutoStrain’s inconsistencies in pediatric patients reflects a limitation in algorithm robustness, underscoring the need for pediatric-specific optimization.

### Limitations

This was a single-center study using only the Philips EPIQ CVx platform with a relatively small sample size, which was unavoidable due to lack of alternate resources. Our small sample size also resulted in an unequal representation of age groups, with the 10–15-year-old group being overrepresented. Moreover, Philips AutoStrain is optimized for Philips-acquired images, whereas TomTec 2DCPA is considered vendor-neutral and can analyze DICOM images from multiple ultrasound manufacturers. Therefore, we limited manual strain analysis to TomTec 2DCPA, as this reflected the tools available at our institution and ensured that the manual analysis method could be applied to vendor-independent datasets. Additionally, the AutoStrain software was only tested on patients in sinus rhythm, as the program relies on electrocardiographic parameters to select ED and ES frames. Therefore, our results cannot be extended to patients with irregular heart rhythms. Our study design intentionally limited inclusion to patients with normal LVEF (> 55%) and structurally normal hearts to minimize confounding variables in this initial validation cohort, which may underestimate the variability encountered in routine clinical practice. Future work should evaluate AutoStrain in patients with abnormal LV size, geometry, and function to better reflect real-world performance. To maintain consistency, all patients were imaged using the X5 transducer, meaning that the X7 probe was not used for our younger patient population, which could affect the result of the present study. A follow-up study using an age-appropriate probe selection would be valuable. In addition, ICC could not be performed on semiautomated Autostrain outputs, as this measurement was performed at bedside during image acquisition by multiple experienced sonographers.

## Conclusions

Our findings indicate that fully automated evaluation of LV function using AutoStrain, while fast and reproducible, is highly dependent on image quality, alignment of the heart, and individual patient characteristics, such as size and heart rate. The measurement variability we observed exceeds thresholds considered clinically acceptable and thus AutoStrain should not be used for large scale analysis or clinical decision making without close operator oversight. These findings should inform both clinicians and developers that without pediatric-specific algorithm training and optimization, the reliability of automated GLS in children remains limited. The current reliance on adult datasets limits the software’s ability to provide accurate and reproducible measurements across the full spectrum of pediatric patients. By addressing these limitations, fully automated programs like AutoStrain could become reliable tools for routine clinical application.

## Supplementary Information

Below is the link to the electronic supplementary material.

Supplementary material 1 (DOCX 12.9 kb)

## Data Availability

Data is provided within the manuscript or supplementary information files.
