# Supplementary material for: Fully Automated Evaluation of Left Ventricular Global Longitudinal Strain in Normal Hearts: An Assessment of Accuracy in the Pediatric Population
Source: Pediatr Cardiol. 2025 Nov 17;47(7):2935–43. doi: 10.1007/s00246-025-04074-2 (PMC13597667; doi:10.1007/s00246-025-04074-2)
Supplement: Supplementary file 1 [file 246_2025_4074_MOESM1_ESM.docx]

**Supplemental Material**

| **Clinical Indication for Echocardiogram** | **Number of Patients**  **N=168** |
| --- | --- |
| Benign cardiac murmur | 55 |
| Follow up for Multisystem Inflammatory Syndrome in Children with preserved LV function | 32 |
| Repaired Tetralogy of Fallot | 21 |
| Pre-chemotherapy (normal LV function) | 10 |
| Mild LV Hypertrophy | 9 |
| Functional Chest Pain | 8 |
| Family history of cardiomyopathy | 8 |
| Aortic Valve Abnormalities | 5 |
| Vasodepressor syncope | 4 |
| Minor coronary artery abnormalities with normal LV function | 4 |
| Post-Arrhythmia Evaluation with normal LV function | 2 |
| Aortic arch anomalies | 2 |
| Suspected mitochondrial Disorder with normal LV function | 2 |
| Hematological Disorder | 2 |
| Partial anomalous pulmonary venous return | 1 |
| Mitral Valve prolapse | 1 |
| Small Patent Ductus Arteriosus | 1 |
| Mild Ebstein’s Anomaly | 1 |
| LV: Left Ventricular |  |

**Supplementary Figure 1.** **Clinical indications for echocardiography in the study cohort**
